# Fetuin-A Characteristics during and after Pregnancy: Result from a Case Control Pilot Study

**DOI:** 10.1155/2012/896736

**Published:** 2012-03-28

**Authors:** Serdar Farhan, Ammon Handisurya, Jelena Todoric, Andrea Tura, Giovanni Pacini, Oswald Wagner, Katharina Klein, Rudolf Jarai, Kurt Huber, Alexandra Kautzky-Willer

**Affiliations:** ^1^3rd Department of Medicine, Cardiology and Emergency Medicine, Wilhelminen Hospital, Montleartstrasse 37, 1160 Vienna, Austria; ^2^Gender Medicine Unit, Division of Endocrinology and Metabolism, Department of Internal Medicine III, Medical University of Vienna, Vienna, Austria; ^3^Metabolic Unit, Institute of Biomedical Engineering, National Research Council, Padova, Italy; ^4^Clinical Institute for Medical and Chemical Laboratory Diagnostic, General Hospital of Vienna, Vienna, Austria; ^5^Department of Obstetrics and Gynecology, Medical University of Vienna, Vienna, Austria

## Abstract

*Objective*. Fetuin-A has been associated with gestational diabetes mellitus (GDM). We investigated fetuin-A levels during and after pregnancy in women with GDM. Fetuin-A measurements were performed in 10 women with GDM and 10 age and body mass index (BMI) matched healthy pregnant women. All women underwent an oral glucose tolerance test (OGTT) in and 3 months after gestation. *Results*. Fasting fetuin-A correlated with BMI in women with former GDM (*r* = 0.90, *P* < 0.0001) but showed no association with parameters of glucose tolerance in women with GDM or post-GDM. GDM featured significantly lower insulin sensitivity and higher insulin and C-peptide secretion profiles compared to NGT during pregnancy (*P* < 0.05). Fasting and postprandial fetuin-A did not differ between groups, neither during nor after pregnancy. *Conclusion*. Fetuin-A is not influenced by glucose tolerance during or after pregnancy or acute glucose elevations following glucose ingestion in young women, but closely relates to BMI early postpartum.

## 1. Introduction

Fetuin-A is a potent systemic calcification inhibitor [1]. Beside this function fetuin-A has been shown to interact with insulin receptor tyrosine kinase, thereby inducing insulin resistance in rodents [2, 3]. In cross-sectional human studies, fetuin-A has been linked to insulin resistance and metabolic syndrome [4]. Furthermore fetuin-A has been linked to incident diabetes mellitus in the Health ABC study [5]. Stefan et al. were able to demonstrate a relation between fetuin-A and liver fat accumulation in insulin-resistant subjects [6]. In pregnant women fetuin-A levels were shown to increase with gestational age [7]. Furthermore women with gestational diabetes (GDM) display higher levels of fetuin-A compared to normal glucose tolerant counterparts [7]. However, GDM being regarded as pre-type 2 diabetes may comprise a very heterogeneous group of women of different ethnicity and with varying degree of obesity. Therefore, investigating fetuin-A behavior in women with GDM may provide a possibility to further elucidate the role of fetuin-A in early stages of diabetes mellitus. Therefore we aimed to investigate the role of acute glucose ingestion during a standardized oral glucose tolerance test (OGTT) on plasma fetuin-A levels in a homogeneous group of European young normal-weight to moderately overweight women with and without GDM during as well as after partum.

## 2. Methods

The study was performed as a case-control study. Women with GDM were recruited consecutively from the Department of Obstetrics and Gynecology of the Medical University of Vienna. Those who gave informed consent underwent a 2 h oral glucose tolerance test (OGTT). Data from 10 women with GDM and 10 women with NGT during pregnancy (28th week of gestation), matched for age and BMI in a 1 : 1 ratio, were analyzed. A second OGTT was performed 10–12 weeks after delivery. Women with previous ketoacidosis and/or beta-cell antibodies (GAD, ICA, IA2), severe chronic disease, kidney or liver disease, and those on chronic medication of drugs known to influence carbohydrate metabolism were excluded.

### 2.1. OGTT

GDM was diagnosed according to the criteria of the 4th International. Workshop Conference on GDM [8]. After an overnight fast for at least 12 h, blood samples for the measurement of glucose, insulin, and C-peptide were taken at baseline as well as at 30, 60, 90, and 120 min after ingestion of 75 g glucose. Fetuin-A levels were measured at fasting as well as 30, 60, and 120 minutes after glucose load.

### 2.2. Plasma Metabolites

Plasma levels of fetuin-A were measured by an enzyme-linked radioimmunoassay (ELISA) (Biovendor laboratory medicine, Modreci, Czech republic). Intra-assay coefficients of variation were 3.5% and interassay coefficient of variation 5.4%. Glucose, insulin, C-peptide, glutamate oxalacetat transaminase (GOT), glutamat-pyruvat-transaminase (GPT), bilirubin, cholinesterase (CHE), cholesterol, high-density lipoprotein cholesterol (HDL-c), low-density lipoprotein cholesterol (LDL-c), triglyceride (TG), and HbA1c were measured using standard kits available in our central laboratory.

### 2.3. Data Analysis

The kinetics of glucose, insulin, and C-peptide during OGTT were analyzed by quantitative methods to obtain metabolic parameters, such as insulin sensitivity through oral glucose insulin sensitivity index (OGIS), which describes glucose clearance per unit change of insulin concentration [9]. Total insulin secretion (TIS) from C-peptide, its suprabasal component (dynamic TIS), and hepatic insulin extraction (HIE) were obtained with a mathematical model of insulin/C-peptide interactions [10, 11]. *β*-Cell function was described as the ability of the *β*-cell to adapt insulin secretion to the prevailing insulin resistance and was quantified by the products: OGIS_dynamic AUC insulin (termed disposition index) and OGIS_dynamic TIS (termed adaptation index), where AUC is the area under the insulin concentration curve during the whole test.

### 2.4. Statistical Analysis

Comparisons of quantitative variables among groups were performed using Student's *t*-test. Associations between continuous variables are described by the Spearman correlation coefficient. Levels of statistical significance were set at *P* < 0.05.

## 3. Results

**Figure 1:**
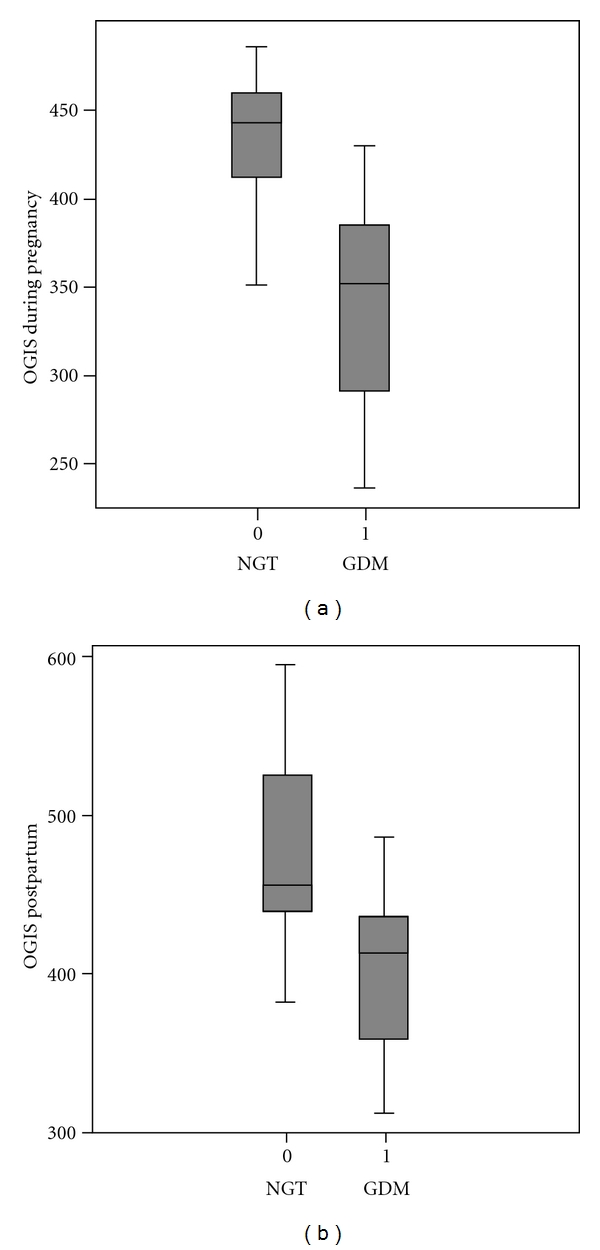
(a) Boxplot of OGIS level (mL min^−1 ^m^−2^) in women with and without gestational diabetes mellitus during pregnancy (*P* = 0.001). (b) Boxplot of OGIS level (mL min^−1 ^m^−2^) in women with and without gestational diabetes mellitus 3 months after delivery (*P* = 0.06).

Women with and without GDM were comparable in terms of their medical history. There were also no differences in baseline as well as 3 months postpartum laboratory parameters of lipoproteins and liver enzymes (Table 1). During gestation women with GDM displayed significant higher fasting insulin, C-peptide, and AUC of both parameters during OGTT. OGIS was significantly lower in women with GDM compared to those with NGT (Table 1) and Figure 1(a).

Three months after delivery, 3 women from the GDM group had a pathologic glucose tolerance status and the remaining 7 returned to normal glucose values. Insulin and C-peptide secretion profiles were still higher in women from the former GDM compared to NGT group but without reaching statistical significance (Table 1). Also OGIS were still lower in the former GDM group compared to women with NGT without statistical significance (Table 1) and Figure 1(b).

**Figure 2:**
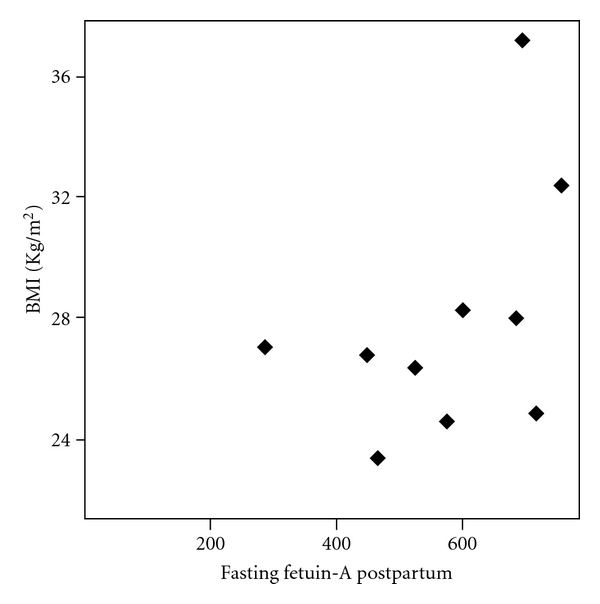
Scatter diagram of the correlation between fasting postpartum fetuin-A (ng/mL) and body mass index (BMI) (Kg/m^2^) in women with history of gestational diabetes mellitus (*r* = 0.90, *P* < 0.0001).

Fasting and postprandial fetuin-A plasma levels did not differ between women with GDM and NGT during as well as 3 months after pregnancy (Table 1). During pregnancy there were no correlations between fetuin-A plasma levels and parameters of insulin resistance and secretion. Three months after gestation there was significant correlation between fasting fetuin-A and BMI (*r* = 0.90, *P* < 0.0001) in women with former GDM (Figure 2). There were no correlations between fetuin-A plasma concentrations and GOT, GPT, or bilirubin.

## 4. Discussion

In the present study we found that fetuin-A plasma levels did not differ between women with and without GDM even when measured during as well as after pregnancy. Additionally we found that fetuin-A was not affected by acute glucose ingestion both during as well as after gestation. Interestingly we found that fetuin-A did not correlate with parameters of obesity and insulin resistance during pregnancy. However three months after gestation there was a strong correlation between fasting fetuin-A and BMI in those from the former GDM group. One study investigated the relation between fetuin-A levels and parameters of insulin resistance during normal pregnancy and in women with GDM [7]. In that study fetuin-A levels increased during the course of uncomplicated gestation [7]. Additionally women with GDM had significantly higher fetuin-A levels compared to healthy pregnant women [7]. Furthermore they found a significant correlation between fetuin-A levels and parameters of insulin resistance such as fasting C-peptide and C-peptide to glucose ratio [7]. However the differences between those results and our findings could be explained by study design and methods. GDM women in the study of Kabaly et al. were more obese than subjects in our investigation. Furthermore we used more sophisticated methods to measure insulin resistance. Under experimental conditions high glucose levels were able to activate fetuin-A gene promoter [12]. This activation was dose dependent and occurred after at least 3 hours of incubation [12]. In our study during OGTT fetuin-A plasma levels remained unchanged. We could not exclude a late elevation of fetuin-A after OGTT. Further studies with longer fetuin-A collection time points during OGTT or intravenous glucose tolerance test are needed to further investigate the influence of glucose per se on fetuin-A plasma concentrations.

However, the fact that even chronic hyperglycemia linked to GDM did not alter fetuin-A levels argues against a prominent effect of glucose fluctuations/elevation on fetuin-A release. Fetuin-A has been linked to obesity in a study performed in patients with morbid obesity [13]. In that study, fetuin-A levels decreased after weight loss due to gastric bypass surgery [13]. Another study investigated the relation between fetuin-A with parameters of insulin resistance and fatty liver [14]. In that study obese children with and without nonalcoholic fatty liver (NAFL) were compared. The authors found a relation between fetuin-A and NAFL as well as a reduction in fetuin-A after weight loss [14]. In our study we did not investigate the presence of NAFL by ultrasound as recommended. Despite this there were no differences in hepatic transaminases as well as cholinesterase between women with and without GDM neither in nor after pregnancy. Additionally we found no correlation of fetuin-A with those liver enzymes (data not presented). Women in our study were only of moderate obesity as compared to children from the investigation of Reinher and Roth [14]. Based on our results and in light of previous findings, it appears that fetuin-A could be the link between fatty liver disease and metabolic syndrome. Further investigations in subjects with obesity with and without NAFL should be performed to finally evidence a link between fatty liver disease, fetuin-A, and insulin resistance.

### 4.1. Study Limitations

First, as we examined a small cohort it is difficult to translate our findings to the general GDM population. Second the subjects investigated were young females, which were besides having GDM healthy. Also the mean BMI of our GDM patients was lower than that of the subjects investigated by Kalabay et al. [7]. Third we did not investigate the presence of NAFL by ultrasound examination.

## 5. Conclusion

Our preliminary study suggests that fetuin-A does not relate to parameters of insulin sensitivity or metabolic control and does not seem to play a role in the pathogenesis of GDM. Furthermore acute glucose changes during an OGTT do not affect fetuin-A plasma concentrations both during and after pregnancy. However preexisting overweight, which may be masked by pregnancy, related changes in body weight or weight retention after pregnancy may have a strong impact on fetuin-A release.

## Figures and Tables

**Table 1:** Patient's characteristics and laboratory parameters.

Variable	Third trimester of gestation		*P*-Value	Three months postpartum		*P* Value
	NGT (*n* = 10)	GDM (*n* = 10)		NGT (*n* = 10)	GDM (*n* = 10)	
BMI (Kg/m^2^)	27.3 ± 1.3	28.9 ± 1.31	0.4	26.5 ± 1.58	27.9 ± 1.30	0.5
Age (years)	30.4 ± 1.7	32 ± 2.3	0.5	—	—	—
Parity	1.1 ± 0.4	2 ± 1	0.4	—	—	—
Systolic blood pressure (mmHg)	116.5 ± 4.2	110 ± 4.1	0.2	117.6 ± 2.9	106.4 ± 4.4	0.05
Diastolic blood pressure (mmHg)	71.5 ± 2.5	67.7 ± 2	0.2	73.1 ± 2.4	65 ± 2.6	0.04
Total cholesterol (mg/dL)	277.1 ± 16.4	227.2 ± 11.6	0.02	204.5 ± 15.2	203 ± 10.2	0.9
LDL cholesterol (mg/dL)	159 ± 14.8	115 ± 9.1	0.02	129.5 ± 13	118.4 ± 6.8	0.4
HDL cholesterol (mg/dL)	75.6 ± 21.3	69.9 ± 3.7	0.4	64.7 ± 6	61.6 ± 4.5	0.6
Triglycerides (mg/dL)	197.1 ± 20.2	207 ± 24.9	0.7	81 ± 7.5	116.3 ± 18.7	0.09
HbA1c (%)	4.9 ± 0.11	4.9 ± 0.14	0.9	5.2 ± 0.1	5.3 ± 0.08	0.5
Cholinesterase ()	5.31 ± 0.71	5.23 ± 1.39	0.6	7.07 ± 0.7	7.76 ± 1.48	0.1
Bilirubin ()	0.33 ± 0.13	0.37 ± 0.14	0.5	0.7 ± 0.42	0.48 ± 0.16	0.1
GOT (U/L)	21.6 ± 5.1	20.1 ± 3.9	0.8	26.3 ± 7.5	26.3 ± 8.9	0.2
GPT (U/L)	15.8 ± 6.1	17.1 ± 7.4	0.4	27 ± 13.7	40.1 ± 23.3	0.9
hs-CRP (mg/dL)	0.43 ± 0.05	2.15 ± 1.75	0.3	0.29 ± 0.07	0.21 ± 0.07	0.5
Fasting glucose (mg/dL)	80.3 ± 2.3	88.1 ± 2.6	0.039	82.4 ± 2.9	92.4 ± 3.6	0.04
1-hour postload glucose (mg/dL)	135.6 ± 7.2	186.1 ± 11.5	0.002	105.3 ± 8.6	114.1 ± 13.4	0.6
Fasting insulin secretion (pmol/L)	12.2 ± 1.6	16.0 ± 1.81	0.1	7.8 ± 1.4	11.3 ± 2.3	0.2
OGIS	434.05 ± 12	341.51 ± 19.35	0.001	476.79 ± 30.23	408.19 ± 18.72	0.06
Disposition index (nmol/m^3^)	3.87 ± 0.44	5.15 ± 0.50	0.07	2.49 ± 0.43	3.57 ± 0.31	0.059
AUC-glucose (mmol/L/min.)	14.21 ± 0.54	19.28 ± 0.73	0.0001	14.17 ± 0.89	17.26 ± 1.26	0.1
AUC-insulin (nmol/L/min.)	8.93 ± 0.98	15.7 ± 2.01	0.007	5.41 ± 1.08	9.11 ± 1.15	0.46
AUC-C-peptide (nmol/L/min.)	1016.7 ± 44.9	1441.3 ± 137.9	0.009	777.5 ± 98.9	1131.4 ± 156.3	0.1
Fasting fetuin-A (ng/mL)	497.5 ± 64.3	580 ± 46.4	0.8	560.38 ± 69.3	571.4 ± 80.4	0.8
30-min. after load fetuin-A (ng/mL)	453.9 ± 29.8	549.3 ± 50	0.5	569.4 ± 86.1	571.3 ± 87.2	0.5
60-min. after load fetuin-A (ng/mL)	581 ± 43.2	513 ± 42.1	0.7	577.5 ± 114.1	542.7 ± 43.8	0.7
120-min. after load fetuin-A (ng/mL)	524.5 ± 39.9	539.6 ± 33	0.1	414.8 ± 48.6	552 ± 47.6	0.1
